# Prevalence and correlates of modifiable and environmental risk factors for non communicable diseases among refugees and asylum seekers in Northern Kenya

**DOI:** 10.1186/s13031-026-00771-8

**Published:** 2026-02-25

**Authors:** Wamalwa Emmanuel, Khamati Sylvia, Ngereso Kevin, Nyoike Martin, Bitok Monicah

**Affiliations:** 1 Daystar University, Nairobi, Kenya; 2 Dansk Røde Kors, Copenhagen, Denmark; 3 Kenya Red Cross Society, Nairobi, Kenya; 4 Ministry of Health, Nairobi, Kenya

**Keywords:** Non-communicable diseases, Refugees, Risk factors, Environmental exposure, Kalobeyei, Kenya, Physical inactivity, Tobacco, Alcohol

## Abstract

**Background:**

Non-communicable diseases (NCDs) are increasingly prevalent in humanitarian settings, yet modifiable and environmental risk factors among displaced populations remain poorly characterized. Refugees in protracted settlements face elevated exposure to risks such as unhealthy diets, physical inactivity, substance use, and indoor air pollution. This study assessed the prevalence and socio-demographic patterns of key NCD risk factors among adult refugees and asylum seekers in the Kalobeyei Integrated Settlement, northern Kenya.

**Methods:**

A cross-sectional mixed-methods study was conducted with 488 adults selected through multi-stage sampling. Structured household surveys assessed modifiable (tobacco and alcohol use, physical activity, diet, salt intake) and environmental (cooking and lighting fuel) NCD risk factors. Bivariate and multivariate logistic regression identified significant associations. Additionally, 18 key informant interviews and 3 focus group discussions were conducted and analyzed thematically.

**Results:**

Nearly all participants (99.6%) reported at least two NCD risk exposures, and 88% had three or more. Lifetime alcohol use was reported by 16.2%, with low education independently associated (OR = 3.51, *p* = 0.017). Tobacco use (6.1%) occurred only among Christians or those with no religion. Only 33.8% met World Health Organization (WHO) physical activity guidelines, with these same groups more likely to be active (OR = 5.82, *p* < 0.001). Low fruit and vegetable intake was widespread, while high salt use (22.8%) was more common among Muslims and older adults. Nearly all households (98.2%) relied on polluting cooking fuels, and over half used polluting lighting sources, disproportionately affecting non-Muslims. Compared to national benchmarks (Kenya STEPwise Survey, 2015), refugees in Kalobeyei reported lower substance use but substantially higher physical inactivity, dietary inadequacy, and environmental exposure. Religious affiliation functioned as a contextual marker for broader sociocultural, gendered, and infrastructural dynamics shaping risk profiles.

**Conclusions:**

Refugees in Kalobeyei experience a high burden of overlapping NCD risk factors, shaped by structural inequalities, service access deficits, and sociocultural context. Addressing these risks requires multisectoral, culturally responsive strategies that integrate health, nutrition, energy, and protection systems in protracted humanitarian settings.

## Introduction

Non-communicable diseases (NCDs) are the leading cause of death globally, accounting for 74% of all deaths [1]. Once considered diseases of affluence, NCDs now disproportionately impact low- and middle-income countries (LMICs), where over 85% of premature NCD-related deaths occur [2]. The primary contributors—cardiovascular diseases, diabetes, cancers, and chronic respiratory illnesses—are driven largely by modifiable and environmental risk factors such as tobacco use, harmful alcohol consumption, unhealthy diets, physical inactivity, and exposure to indoor air pollution [3, 4]. Without urgent and sustained preventive action, global projections forecast a continued rise in NCD mortality, undermining Sustainable Development Goal (SDG) 3.4, which aims to reduce premature NCD deaths by one-third by 2030 [2].

Although NCDs have gained attention in global policy arenas, prevention efforts in humanitarian contexts remain limited [5, 6]. Refugees and asylum seekers are at heightened risk of NCDs due to disrupted livelihoods, food and energy insecurity, overcrowded living conditions, psychosocial stress, and limited access to healthcare [7, 8]. For example, food insecurity in camps often results in reliance on low-diversity, calorie-dense diets, while economic hardship fuels increased use of alcohol and tobacco [9, 10]. Furthermore, over 90% of forcibly displaced households in LMICs rely on biomass fuels such as charcoal and firewood for cooking, leading to harmful levels of indoor air pollution that contribute to respiratory and cardiovascular disease [4, 11]. Yet humanitarian health responses continue to prioritize communicable disease control and acute care, leaving the NCD prevention agenda largely neglected [12].

There is now growing concern over the emerging burden of modifiable and environmental NCD risk factors in refugee settings. Recent studies report rising use of tobacco and alcohol, widespread physical inactivity, and inadequate fruit and vegetable consumption among displaced populations [13]. Displaced women are especially affected, facing sociocultural barriers that limit their ability to engage in physical activity, including safety concerns, restrictive norms, and caregiving burdens [14]. In many camps and settlements, individuals are exposed to multiple concurrent risk factors, leading to syndemic health effects that exacerbate chronic disease outcomes [15]. In East Africa, more than 70% of displaced households have been shown to face three or more NCD risk factors simultaneously [16].

Sub-Saharan Africa is at the epicenter of this NCD transition. The region faces rising NCD morbidity and mortality, compounded by weak health systems and underinvestment in prevention [17]. In Kenya, the burden of NCDs has grown sharply, accounting for nearly 40% of all annual deaths, up from 27% in 2014 (Kenya Ministry of Health [MoH], 2022). Many NCDs affect people in their productive years, intensifying cycles of poverty and dependence. The Horn of Africa, including Somalia, South Sudan, and Ethiopia, grapples with similar challenges, where fragile political and health infrastructures further constrain NCD care and prevention [14].

In Kenya, the majority of refugees reside in Turkana County’s Kakuma Refugee Camp and the adjacent Kalobeyei Integrated Settlement. Established in 2015, Kalobeyei was designed to promote self-reliance and integrate refugee and host communities through shared services and infrastructure. As of May 2025, the settlement hosted approximately 80,000 refugees and asylum seekers, primarily from South Sudan, Somalia, and the Democratic Republic of Congo. Despite its development-oriented design, Kalobeyei continues to face deep challenges—including heavy dependence on humanitarian cash transfers, limited dietary diversity, and poor access to clean energy—factors that elevate the risk of both modifiable and environmental NCD exposures.

Although recent evaluations in similar humanitarian contexts point to widespread NCD risk factors, including sedentary lifestyles, poor diet, substance use, and indoor pollution [16, 18], existing research remains largely focused on access to treatment and continuity of care. Little attention has been paid to upstream modifiable and environmental determinants [17], Brolin Ribacke, Saulnier, Eriksson, & von Schreeb, 2016). Moreover, most of the evidence originates from Middle Eastern refugee contexts, such as among Syrian populations in Jordan and Lebanon, which may not reflect the sociocultural and environmental realities of East African settings [13], Hajjar, Atun, & Kieny, 2021). In Kenya specifically, there is a marked lack of empirical data on NCD risk prevalence and their socio-demographic correlates among displaced populations, despite the country hosting one of the largest refugee populations in the region.

Addressing NCDs in protracted refugee settings requires a shift from treatment-focused models to prevention-oriented, multi-sectoral approaches. Interventions targeting modifiable risk behaviors, such as tobacco and alcohol use, poor diets, and physical inactivity, must be integrated with strategies for food security, social protection, clean energy, and health literacy [19], Peters et al., 2015). Designing such integrated responses, however, demands robust, context-specific evidence on which groups are most at risk and how exposures vary by age, sex, religion, and education.

This study responds to that evidence gap by examining the prevalence and socio-demographic correlates of key modifiable and environmental risk factors for NCDs among adult refugees and asylum seekers in Kalobeyei. Specifically, it assesses alcohol and tobacco use, physical inactivity, inadequate fruit and vegetable intake, high salt consumption, and exposure to polluting household fuels for cooking and lighting. Through a mixed-methods design, the study not only quantifies risk prevalence but also provides qualitative insights into the structural and cultural factors shaping health behavior.

Ultimately, NCDs present an urgent but under-recognized challenge in protracted displacement settings. Refugees in Kalobeyei face multiple, intersecting exposures to modifiable risk factors that, if left unaddressed, will compound existing health inequities and undermine long-term wellbeing. Generating and acting upon locally relevant data is essential to ensure that displaced populations are not left behind in the global drive for universal health coverage and the realization of SDG 3.4.

**Conceptual framework:** This study is guided by the WHO framework on modifiable and environmental risk factors for non-communicable diseases, situated within a social determinants of health and syndemic perspective. Behavioral risk factors (tobacco use, alcohol consumption, physical inactivity, unhealthy diet, and high salt intake) and environmental exposures (household cooking and lighting fuels) are conceptualized as upstream determinants of NCD risk that are shaped by broader socio-demographic, cultural, and structural conditions in protracted displacement settings. Socio-demographic characteristics such as age, sex, education, and religious affiliation are treated as contextual markers that may reflect underlying norms, resource access, and living conditions, rather than as direct causal mechanisms. This framework informed the selection of study variables and guided interpretation of observed associations.

## Materials and methods

### Study design

This study adopted a descriptive cross-sectional design to estimate the prevalence and examine socio-demographic correlates of modifiable and environmental risk factors for NCDs among adult refugees and asylum seekers residing in Kalobeyei Integrated Settlement, Turkana County, Northern Kenya. A convergent mixed-methods approach was employed, combining structured household surveys with Key Informant Interviews (KIIs) and Focus Group Discussions (FGDs). Quantitative data were used to quantify risk factor prevalence and assess socio-demographic associations, while qualitative data were collected and analyzed to contextualize risk behaviors, explore underlying drivers of observed patterns, and interpret quantitative findings, particularly in relation to lifestyle behaviors, environmental exposures, health-seeking behavior, and health system constraints.

### Study context

Kalobeyei Integrated Settlement hosts a protracted refugee population of approximately 79,685 individuals primarily from South Sudan, DRC, Somalia, and Ethiopia. Unlike traditional camps, Kalobeyei promotes a development-oriented humanitarian approach, integrating host and refugee communities through shared services and livelihood promotion. The Continuity of NCD Care in Crisis (CIC) programme, implemented by the Danish Red Cross and Kenya Red Cross Society with support from the Novo Nordisk Foundation since 2022, aims to enhance sustained access to NCD prevention and care services in the settlement. This study contributes to the interim evaluation of CIC, focusing specifically on modifiable (e.g., alcohol use, smoking, physical inactivity, diet) and environmental (e.g., cooking and lighting fuel use) risk factors for NCDs.

### Sampling and study population

The household survey involved 488 adult respondents selected using multi-stage cluster sampling across four settlement zones: Village 1, Village 2, Village 3, and the reception center. Sample size was calculated using Yamane’s formula (1967) with a 95% confidence level, 5% precision, and 20% non-response buffer. Within each zone, 30% of residential compounds were randomly selected. Proportionate stratified sampling was applied within compounds to allocate respondents based on the number of households. In each selected household, one adult (≥ 18 years) was randomly chosen using a simple listing method. Substitutions were made only if the selected respondent was unavailable after repeated visits. In addition, 18 KIIs were conducted with national and county-level health managers, NCD officers, UNHCR officials, and NGO partners, while three FGDs were held with Community Health Promoters (CHPs), male NCD support group members, and female support group members respectively.

### Data collection instruments and procedures

- **Household survey questionnaire**: Quantitative data were collected using a structured questionnaire covering socio-demographics, knowledge of NCDs, and key modifiable and environmental risk factors (alcohol and tobacco use, dietary behavior, physical activity, cooking and lighting fuel). The tool also assessed exposure patterns and access to health information. Trained enumerators administered the questionnaire face-to-face using KoBo Toolbox on tablets.
- **KII guide**: Designed to collect expert views on service delivery, NCD burden, policy gaps, and structural challenges in the humanitarian context. Respondents included NCD coordinators, public health officers, and humanitarian actors engaged in CIC implementation.
- **FGD guide**: Explored perceptions, social norms, risk behaviors, and lived experiences related to NCDs and service uptake. Each FGD consisted of 8–10 participants, conducted in local languages by trained facilitators and note-takers.

All tools were pre-tested in Kakuma Refugee Camp, and revised for clarity and contextual relevance. Data collectors received two days of training, including sessions on consent procedures, digital data collection, probing techniques, and managing sensitive disclosures. Field protocols emphasized dignity, privacy, and respondent safety.

### Data analysis

**Quantitative analysis**: Data from KoBo Toolbox were cleaned and analyzed using STATA. Descriptive statistics (frequencies, means, percentages) were used to summarize participant characteristics and prevalence of risk behaviors. Bivariate associations were assessed using chi-square tests (p < 0.05). To identify independent socio-demographic correlates of selected risk factors (e.g., alcohol use, physical inactivity, vegetable intake, salt use), binary logistic regression models were fitted with adjusted odds ratios (AORs) and 95% confidence intervals (CIs) reported.

Multivariable logistic regression was applied to outcomes that demonstrated sufficient variability and adequate event counts, and where modelling was theoretically and statistically appropriate. Outcomes with near-universal prevalence, highly skewed distributions, or limited independent variation after bivariate screening were not modelled using multivariable regression, as such analyses would yield unstable or non-informative estimates.

Covariate selection for multivariable models followed a theory-informed and context-driven approach. Socio-demographic variables were selected a priori based on established frameworks for NCD risk (including age, sex, education, and religion) and prior evidence from humanitarian settings and were retained to control for potential confounding. Additional covariates were considered for inclusion based on bivariate associations (*p* < 0.10) and assessment of model stability. This combined approach was adopted to balance theoretical relevance with empirical support while avoiding overfitting in a sample with limited event counts for some outcomes. In models with sparse data or limited event counts, inclusion of all a priori covariates (including age and sex) was not always feasible due to risks of model non-convergence or unstable estimates, and final model specifications reflect these statistical constraints rather than theoretical irrelevance. For some outcomes with sparse data or perfect separation, multivariable estimates were unstable, resulting in wide confidence intervals; these results were therefore interpreted cautiously and emphasized descriptively rather than inferentially.

**Qualitative analysis**: In this study, qualitative findings across community, facility, and programme stakeholders provided explanatory depth to these patterns, particularly by illuminating how structural deprivation, sociocultural norms, and service constraints shape observed NCD risk behaviors. KII and FGD transcripts were analyzed thematically using NVivo software. Coding frameworks were developed deductively from research objectives and inductively from emerging themes. Matrices were used to compare responses across stakeholder groups. Themes included structural barriers, gendered risk behaviors, faith-based influences, and intervention gaps.

**Mixed-methods integration**: Triangulation of quantitative and qualitative data was conducted during analysis and interpretation to validate findings and strengthen conclusions. For instance, religious influences on alcohol and tobacco use observed in survey data were contextualized by qualitative narratives from CHPs and faith leaders.

Findings were interpreted relative to available national benchmarks, particularly the Kenya STEPwise Survey for NCDs Risk Factors [37] to situate refugee risk profiles within the broader Kenyan context.

## Results

### Demographics

The survey captured responses from 488 adults, with a significant majority being female (73.8%), which is reflective of household structures and daytime availability during data collection. The age distribution skewed young, with 63.1% of respondents aged between 18 and 34 years. A substantial proportion (36.1%) reported having no formal education, highlighting potential vulnerabilities in health literacy and information access. The dominant source of livelihood was humanitarian assistance (66.2%), with very few respondents engaged in stable or formal income-generating activities Table 1.

**Table 1:** Respondents demographic characteristics

Demographic characteristics		Total % (n = 488)
Sex	Female	73.8%
	Male	26.2%
Highest level of Educational attainment	None	36.1%
	Primary	25.2%
	Secondary	35.7%
	Tertiary	2.5%
	Madarasa	0.6%
Marital Status	Married/living together as if married	53.5%
	Single	29.7%
	Separated	8.4%
	Widowed	6.1%
	Divorced	2.3%
Age	18–24 years	33.4%
	25–34 years	29.7%
	35–44 years	21.9%
	45–54 years	12.1%
	55–64 years	2.7%
	> 65 years	0.2%
Religious Affiliation	Christian	85.7%
	Muslim	13.5%
	No religion	0.8%
MAIN Source of household income	Cash transfers from UNHCR/other agencies	66.2%
	Formal employment	5.5%
	Business	1.6%
	Farming	1.2%
	Remittances from kin	0.8%
	Others	24.6%

This study examined modifiable and environmental risk factors for NCDs among refugees and asylum seekers living in Kalobeyei Integrated Settlement, in Northern Kenya. The results indicate a population with widespread and overlapping exposures—including alcohol and tobacco use, physical inactivity, unhealthy diets, high salt consumption, and reliance on polluting household fuels. These clustered risks substantially increase vulnerability to chronic disease, with patterns of exposure varying across key sociodemographic characteristics such as education, religion, age, and sex. It is important to note that all associations presented below are interpreted as correlations, not causal mechanisms.

### Alcohol use

Alcohol use was relatively common among respondents in Kalobeyei. Overall, 16.2% (79/488) reported lifetime alcohol consumption, and nearly half of these (48.1%, 38/79) were current drinkers. Drinking frequency varied, with 39.5% reporting weekly consumption, 18.4% daily use, and 34.2% monthly use. Traditional brews dominated consumption patterns: chang’aa^1^ (55.3%) and busaa^2^ (47.4%) were most commonly consumed, while commercial beverages such as beer (7.9%) and wines/spirits (5.3%) were less prevalent.

Markers of harmful alcohol use were evident. Among current drinkers, 47.4% reported failing to meet responsibilities due to drinking at least sometimes in the past year (31.6% “sometimes”; 15.8% “very often”). Similarly, 52.6% reported needing a first drink in the morning after heavy drinking (39.5% “sometimes”; 13.2% “very often”). Encouragingly, 31.6% of current drinkers reported receiving some form of support to quit, indicating partial access to social or health system interventions for alcohol misuse. Qualitative interviews with community health promoters and programme staff described alcohol use as coping responses to psychosocial stress and economic hardship in the settlement.

Bivariate and multivariable analyses identified key socio-demographic correlates of alcohol use. Low educational attainment was associated with current alcohol use in both bivariate and adjusted analyses. Respondents with no or only primary education were more likely to report current alcohol use compared to those with secondary or higher education (χ^2^(1) = 4.80, p = 0.028), and this association remained significant after adjustment (OR = 3.51, 95% CI: 1.25–9.84, p = 0.017). Lifetime alcohol use was strongly associate with religious affiliation, with 97.5% (77/79) of ever drinkers being non-Muslim (χ^2^(1) = 28.1, p < 0.001). However, after adjustment, religious affiliation was not a statistically significant predictor of current alcohol use (OR = 1.36, 95% CI: 0.08–23.01, p = 0.830). No significant associations with current alcohol use were observed by sex, age group, or marital status (p > 0.05). It should be noted that religious affiliation should be interpreted as reflecting broader sociocultural norms, gender roles, and settlement dynamics, rather than religionQuery itself acting as a direct causal determinant of risk behaviors Table 2.

**Table 2:** Logistic regression of correlates of current alcohol use

Variable	OR	95% CI (Lower–upper)	p-value
Low education	3.51	1.25 – 9.84	0.017
Non-Muslim	1.36	0.08 – 23.01	0.830

### Tobacco use

Overall, 6.1% (30/488) of respondents reported ever smoking tobacco products such as cigarettes, cigars, pipes, or shisha. Of these, 40% (n = 30) self-reported as having had smoked in the 30 days preceding the study, representing 2.5% of the total sample being active smokers. Among those who had ever smoked, cigarettes were the most common product (30.0%), followed by shisha (20.0%), cigars (6.7%), pipes (6.7%), and 3.3% Atamgala (Ethiopian tobacco). Among those who had smoked within 30 days preceding the study, the distribution remained similar, with cigarettes (41.7%) dominating current use, followed by shisha (25.0%), pipes (16.7%), cigars (8.3%), and other products (16.7%). Quit attempts were relatively common: 66.7% of current smokers reported having tried to stop smoking in the past year, although most remained active users at the time of the survey. Only 0.8% of the total population reported currently using smokeless tobacco products, such as snuff, chewing tobacco or betel. Similar to alcohol, qualitative interviews with community health promoters and programme staff described tobacco use as coping responses to psychosocial stress and economic hardship in the settlement.

Bivariate and multivariable analyses identified limited but distinct socio-demographic patterns in tobacco use. Ever smoking was more common among respondents with lower education compared to those with secondary or higher education (7.0% vs. 4.3%), though this association did not reach conventional statistical significance (χ^2^(2) = 5.34, p = 0.069). All ever smokers were non-Muslim, yielding a statistically significant bivariate association with religious affiliation (χ^2^(1) = 3.84, p = 0.050). Smoking prevalence did not differ significantly by sex, refugee status, settlement zone, age group, or marital status (p > 0.05). In multivariable logistic regression, religious affiliation emerged as the only predictor of tobacco use; however, estimates were unstable due to perfect separation, as no tobacco use was reported among Muslim respondents. Educational attainment and sex were not statistically significant predictors after adjustment. It should be noted that religious affiliation should be interpreted as reflecting broader sociocultural norms, gender roles, and settlement dynamics, rather than religion itself acting as a direct causal determinant of risk behaviors.

### Physical inactivity

Physical inactivity emerged as an important modifiable risks in Kalobeyei. Overall, only 33.8% of respondents met the WHO recommended guidelines for physical activity (≥ 150 min of moderate-intensity or ≥ 75 min of vigorous-intensity activity per week). This indicates that two-thirds of the population were insufficiently active, placing them at increased risk of NCDs.

Bivariate and multivariable analyses identified religious affiliation as the primary socio-demographic correlate of physical activity. Men were slightly more likely than women to meet WHO physical activity guidelines (36.2% vs. 32.4%), though this difference was not statistically significant (χ^2^(1) = 2.47, p = 0.116). Respondents with secondary or higher education also showed marginally higher activity levels compared to those with no or primary education (35.7% vs. 32.8%), but this association was not significant (χ^2^(2) = 2.08, p = 0.353). Physical activity levels varied significantly by religious affiliation, with non-Muslims more likely to meet WHO recommendations (χ^2^(1) = 19.58, p < 0.001). Physical activity declined with age, although this trend was not statistically significant (χ^2^(3) = 3.69, p = 0.297). It is important to note that the study population was predominantly young, with 93.6% of respondents aged below 50 years and a mean age of 31.7 years. In multivariable logistic regression, religious affiliation remained the only independent predictor of sufficient physical activity. Non-Muslim respondents had significantly higher odds of meeting WHO physical activity guidelines compared to Muslim respondents (OR = 5.82, 95% CI: 2.45–13.81, p < 0.001). Other demographic variables were not statistically significant predictors after adjustment (p > 0.05). It should be noted that religious affiliation should be interpreted as reflecting broader sociocultural norms, gender roles, and settlement dynamics, rather than religion itself acting as a direct causal determinant of risk behaviors. Qualitative participants reported safety concerns, gendered household roles, and limited recreational space as common barriers to regular physical activity, particularly among women.

### Diet and salt intake

Knowledge of balanced diet**:** Overall, 58.2% of respondents correctly identified components of a balanced diet, most commonly proteins, carbohydrates, and vitamins. Fewer respondents mentioned minerals, fiber, or water.

Fruit and vegetable intake**:** Fruit consumption was uniformly low, with 99.2% of respondents consuming fruits on fewer than five days per week, and no significant differences observed by education, sex, or religious affiliation (p > 0.05). Vegetable intake was also suboptimal, with 72.4% of respondents consuming vegetables on fewer than five days per week. Inadequate vegetable intake varied significantly by religious affiliation, with non-Muslims more likely to report low intake compared to Muslims (76.8% vs. 51.5%, χ^2^(1) = 17.37, p < 0.001). This association remained significant after adjustment, with non-Muslims having higher odds of inadequate vegetable intake (OR = 3.01, 95% CI: 1.76–5.16, p < 0.001). Education level and sex were not significant predictors.

Salt intake**:** Overall, 22.8% of respondents reported high salt intake, defined as adding salt at the table daily and/or consuming processed foods high in salt daily, consistent with the Kenya STEPwise Survey 2015 definition. High salt intake varied significantly by religious affiliation and age. Muslim respondents reported a higher prevalence of high salt intake compared to non-Muslims (37.9% vs. 22.0%; χ^2^(1) = 6.97, p = 0.008). Salt intake increased with age, from 17.2% among respondents aged 18–24 years to 37.0% among those aged ≥ 45 years (χ^2^(3) = 12.58, p = 0.006). Differences by sex and education were not statistically significant (p > 0.05).

### Environmental risk factors

Cooking fuel**:** Use of polluting fuels was nearly universal, with 98.2% of households relying on charcoal, firewood, or kerosene. Clean fuels such as LPG, electricity, or solar were used by only 1.8%. Due to the near-universal prevalence of polluting fuel use, no meaningful statistical associations with socio-demographic factors were observed. Qualitative data underscored economic and infrastructural barriers: *“Even if gas is safer, most can’t afford it. Firewood is what we have”* (KII, Host Community Leader).

Lighting fuel**:** Lighting sources were more variable: 53.3% of households used polluting sources (kerosene, candles, firewood), while 46.7% used cleaner options (solar, electricity). Use of polluting lighting was significantly higher among non-Muslims (χ^2^(1) = 39.42, p < 0.001), a pattern confirmed in logistic regression (OR = 7.66, 95% CI: 3.87–15.17, p < 0.001). These associations may reflect underlying socio-spatial or infrastructural differences patterned by religious affiliation, including structural and spatial inequalities, such as settlement layout and access to solar installations, rather than religious affiliation per se. As noted in FGDs: *“The newer zones have solar; others still use kerosene depending on where you were settled.”* Other demographic variables were not significant. Overall, while access to clean lighting is improving, clean cooking energy remains virtually absent. Environmental exposures are shaped by affordability and infrastructure gaps, requiring targeted interventions to expand equitable access.

### Composite risk burden

Exposure to multiple NCD risk factors was universal. All respondents reported at least two overlapping risks, and 88.6% had three or more. This clustering was driven by widespread use of polluting cooking fuels and inadequate fruit and vegetable intake, alongside high salt use, physical inactivity, unsafe lighting, and smaller but notable proportions reporting alcohol and tobacco use.

Due to the highly skewed distribution, the composite risk burden was summarized descriptively. Multivariable analysis was not pursued, as limited variation would have produced unstable or uninformative estimates. Qualitative data reinforced these patterns, highlighting intersecting stressors, poverty, displacement, food insecurity, and limited services, that compound household-level NCD risk.

## Discussion

### Overview of key findings

This study documents a high prevalence and clustering of modifiable and environmental NCD risk factors among adult refugees and asylum seekers in Kalobeyei Integrated Settlement. All participants reported at least two risk exposures, with the majority facing three or more. The most common patterns included inadequate fruit and vegetable intake, physical inactivity, high salt consumption, substance use, and near-universal reliance on polluting cooking fuels. These exposures varied across socio-demographic lines, particularly education, age, and religious affiliation, suggesting that NCD vulnerability is patterned by broader sociocultural and structural dynamics within the displacement context. The following discussion explores these patterns in depth, drawing on both quantitative and qualitative findings to examine how intersecting vulnerabilities shape health behaviors in humanitarian settings.

### Demographic vulnerabilities and NCD risk

The study population was predominantly female (73.8%) and young (63.1% under 35 years), with over one-third having no formal education and two-thirds dependent on humanitarian aid. These findings underscore the intersecting vulnerabilities faced by displaced populations in accessing and acting on NCD-related information. Previous research in similar settings has shown that gendered roles and limited educational attainment can hinder health literacy and delay care-seeking, especially for asymptomatic chronic conditions [13, 20]. The socio-economic dependence on aid suggests constrained agency in adopting preventive behaviors, especially those linked to cost, such as dietary diversity and clean energy use.

### Substance use: alcohol and tobacco

**Alcohol use** in Kalobeyei was relatively common, with 16.2% of respondents reporting lifetime consumption and nearly half being current users. Traditional brews such as *chang’aa* and *busaa* dominated drinking patterns, reflecting informal alcohol economies that thrive in settings marked by poverty, displacement, and limited enforcement [21]. Indicators of harmful use, such as morning drinking and disruption of responsibilities, were frequently reported, underscoring the potential for dependence even within a small user group. Low educational attainment emerged as an independent predictor of current alcohol use, consistent with global and regional evidence linking low socioeconomic status to elevated substance use risks [22, 23]. Qualitative data echoed this, with alcohol framed as a coping mechanism for psychosocial stress, trauma, and unemployment. Although religious affiliation was strongly associated with lifetime alcohol use in bivariate analysis, it did not remain significant after adjustment, suggesting that socio-spatial and cultural dynamics may be more relevant than religion alone.

**Tobacco use** was less prevalent overall, 6.1% reported ever smoking, and only 2.5% were active smokers. Among those currently using tobacco, cigarettes and shisha were the most commonly used products. Notably, over two-thirds of current smokers had attempted to quit in the past year, yet most remained active users. This indicates partial motivation for cessation but limited access to effective support systems. As with alcohol, qualitative accounts described tobacco use as a response to economic hardship and psychosocial distress in the settlement. Religious affiliation was significantly associated with tobacco use in bivariate and adjusted analyses; all smokers were non-Muslim. However, this pattern should be interpreted cautiously due to perfect separation in the data and the potential role of religion as a proxy for broader settlement-level or cultural differences. Education and sex were not significant predictors, possibly due to limited variation and small sample size. Although tobacco use was less common than alcohol use, its presence in high-stress, underserved environments, coupled with limited cessation support, suggests a potential for normalization and progression over time, particularly among younger or unemployed adults.

When compared to national data from the Kenya STEPwise Survey (MoH, 2015), which reported alcohol and tobacco use among 19% and 13% of adults respectively, both behaviors were less prevalent in Kalobeyei. However, this may reflect restricted access, religious and cultural norms, and lower purchasing power, rather than reduced vulnerability. The presence of harmful use indicators, even at low prevalence, highlights the need for integrated, trauma-informed substance use interventions within NCD prevention frameworks for refugee populations.

### Physical inactivity

Physical inactivity was highly prevalent, with only one-third of respondents meeting WHO guidelines, substantially higher than the 7% national estimate reported in the Kenya STEPwise Survey (MoH, 2015). This elevated risk profile is particularly concerning given co-existing dietary risk factors and limited access to NCD screening or care in refugee settings. Religious affiliation was the only significant predictor of physical activity after adjustment, with Muslim respondents significantly less likely to meet activity recommendations. This association likely reflects structural and cultural constraints rather than religious doctrine per se. Qualitative data pointed to intersecting barriers, including restrictive gender norms, safety concerns, and limited recreational space, especially for women, consistent with findings from other conservative or crisis-affected settings [24–26]. Although age, sex, and education were not significant predictors in this population, the role of gendered household roles, mobility restrictions, and fear of harassment were recurrent themes in FGDs and KIIs. For example, one participant noted: *“You cannot just go out walking as a woman, it’s not safe, and people talk.”* These insights highlight that promoting physical activity in such settings requires more than individual motivation,it demands attention to environmental design, protection concerns, and cultural sensitivity. To reduce sedentary behavior and mitigate long-term NCD risk, integrated interventions are needed that embed safe and inclusive opportunities for movement, particularly for women and youth, within broader settlement planning, protection programming, and health promotion efforts.

### Diet, salt intake, and nutrition knowledge

Fruit and vegetable intake was strikingly inadequate. Nearly all respondents (99.2%) consumed fruits on fewer than five days per week, while 72.4% consumed vegetables below this threshold, far below WHO dietary recommendations [27]. Low fruit intake showed no variation by sex, education, or religion, indicating a systemic issue likely rooted in food insecurity, limited access to fresh produce, and reliance on food aid [28], FAO, 2017). Vegetable consumption, however, was associated with religious affiliation: non-Muslims had significantly lower intake than Muslims, even after adjusting for covariates. This may reflect cultural or religious dietary norms, such as emphasis on fresh food in Muslim meal patterns [29]. Still, religion likely functions as a proxy for broader sociocultural or infrastructural differences, such as access to markets or cooking practices, an interpretation we caution throughout. Although 58.2% of respondents could identify key components of a balanced diet, fewer mentioned minerals, fiber, or water. This partial knowledge did not translate into healthier eating behaviors, highlighting the limits of awareness-based interventions. Broader constraints, economic, logistical, and cultural, shape dietary behavior in refugee settings [30, 31]. Salt intake was elevated in 22.8% of respondents, with significantly higher rates among Muslims (37.9%) and older adults (37.0% in ≥ 45 years). These trends mirror global evidence linking salt preference with cumulative dietary exposure and aging [32, 38]. While Muslim participants had lower risk profiles in other domains (e.g., tobacco, alcohol, inactivity), their higher salt intake suggests divergent household practices in food preparation or seasoning, warranting further inquiry.

Compared to national data from the Kenya STEPwise Survey (MoH, 2015), where 94% of adults consumed insufficient fruits/vegetables and 26% had high salt intake, Kalobeyei exhibits greater deficiency in fresh produce and comparable salt exposure. This underscores the compounded nutritional risks faced by displaced populations. Overall, the findings reveal a double burden: pervasive under-consumption of protective foods, and elevated intake of harmful ones like salt. Religious affiliation was a significant, though complex, correlate. Interventions must therefore go beyond health education, addressing underlying constraints through culturally-sensitive behavior change, diversified food assistance, and improved access to fresh produce. Faith-informed approaches and gender-responsive strategies, integrated into existing humanitarian delivery channels, can offer scalable entry points for nutritional risk reduction.

### Environmental exposures

Environmental determinants of NCDs remain critically under-addressed in humanitarian contexts. In Kalobeyei, over 98% of households relied on polluting fuels for cooking, and more than half used polluting sources for lighting, exposures linked to chronic respiratory illness, cardiovascular disease, and adverse maternal outcomes [39]. These findings point to entrenched energy poverty, especially in cooking fuel, where clean energy access was virtually nonexistent. Religious affiliation was associated with lighting fuel type, with non-Muslims more likely to rely on polluting sources. However, this likely reflects deeper socio-spatial and infrastructural differences, such as settlement location, housing quality, or access to solar installations, rather than faith-based behaviors per se. As noted in FGDs, newer housing blocks had better solar access, while older areas lacked such infrastructure.

Unlike other modifiable risk factors, household energy use is shaped less by individual knowledge or behavior and more by structural and infrastructural constraints. The absence of clean cooking fuels across the board underscores systemic neglect of energy transitions in refugee planning and financing. While national surveys such as the Kenya STEPwise Survey (MoH, 2015) do not systematically assess household energy use, the scale of reliance on biomass in Kalobeyei likely exceeds national averages, amplifying NCD risks among displaced populations.

Addressing these exposures will require integrated, multi-sectoral approaches, linking health, energy, and settlement infrastructure, rather than stand-alone health messaging. Expanding access to affordable, clean energy must become a priority within humanitarian resilience and NCD prevention strategies.

### Composite risk burden

The finding that all respondents reported at least two NCD risk factors, and nearly 88% had three or more, reflects a syndemic pattern of clustered exposures shaped by structural vulnerability [15]. This aligns with global evidence that NCD risks in humanitarian settings co-occur and interact, rather than appear in isolation. In Kalobeyei, the most prevalent risk factors, polluting cooking fuels, inadequate diet, high salt intake, physical inactivity, and unsafe lighting, are not merely behavioral but embedded in systemic constraints. These overlapping exposures cannot be addressed through siloed health interventions. For example, physical activity is limited not only by individual motivation, but by gendered mobility restrictions, safety concerns, and a lack of supportive infrastructure. Similarly, efforts to improve dietary practices must engage with food affordability, market access, and humanitarian food assistance models that prioritize quantity over quality. High reliance on polluting fuels reflects deficits in energy infrastructure rather than energy preferences.

Several mediation pathways underlie these risk profiles. For instance, gender norms and household roles influence mobility, time use, and opportunities for physical activity; food access constraints are shaped by humanitarian assistance modalities, income limitations, and market availability; socio-spatial inequality shapes energy source availability; and deficits in energy infrastructure constrain reliance on clean cooking and lighting options. These factors interact to produce cumulative, context-driven risk burdens at household level, underscoring that most observed associations operate through structural, not individual-level, mechanisms.

Addressing this composite risk burden requires integrated, multi-sectoral approaches that cut across health, nutrition, energy, and protection systems. NCD prevention in humanitarian contexts must move beyond awareness campaigns to structural interventions that expand access, reduce barriers, and improve living conditions.

### Implications for humanitarian programming

These findings highlight the need for multi-sectoral, structural approaches to NCD prevention in refugee settings. Risk factors in Kalobeyei, such as poor diet, physical inactivity, and polluting fuel use, are shaped by gender norms, food access constraints, socio-spatial inequalities, and infrastructure deficits. As such, interventions must move beyond awareness campaigns to address the underlying conditions driving risk exposure. NCD prevention should also be integrated across diverse humanitarian platforms, not limited to Community Health Promoters. Health promotion efforts can be embedded in nutrition, education, protection, and livelihood programs. Food assistance modalities should be reoriented to improve access to fruits and vegetables and reduce dependence on salt-heavy staples, using market-based approaches where feasible. Gender-responsive infrastructure, including safe spaces for physical activity, is essential to overcome mobility and safety barriers, especially for women and youth. Finally, addressing energy poverty, particularly the near-universal use of polluting cooking fuels, requires investment in clean energy infrastructure and subsidies, recognizing its role in long-term NCD prevention. Overall, humanitarian actors should adopt an integrated model that tackles the social, environmental, and service-level determinants of NCD risk, ensuring displaced populations are not left behind in efforts toward health equity and universal health coverage.

### Study limitations

- Measurement of behavioral risk factors relied on self-reported data, which may be subject to recall bias and social desirability bias, particularly for sensitive behaviors such as alcohol and tobacco use. These biases may have led to under-reporting, especially in contexts where substance use is socially or religiously discouraged. However, self-report remains the most feasible and widely used approach for population-level assessment of NCD risk behaviors in humanitarian settings, including in the WHO STEPwise framework. To mitigate bias, standardized questionnaires were used, interviews were conducted by trained data collectors in private settings, and culturally appropriate phrasing was employed to reduce judgment and encourage accurate reporting.
- Dietary intake and salt consumption were assessed using proxy indicators rather than objective or biochemical measures. High salt intake was defined based on reported behaviors such as frequent addition of salt at the table and consumption of processed salty foods, consistent with the Kenya STEPwise Survey methodology. While these proxies do not capture actual sodium intake, they are widely used in population-based surveys and humanitarian settings where 24-h urinary sodium collection or detailed dietary recalls are not feasible. As such, findings related to salt and diet should be interpreted as indicative of risk-related behaviors rather than precise estimates of intake.
- Small cell sizes in some subgroup analyses (e.g., Muslim drinkers or smokers) reduced statistical power and limited the stability of adjusted estimates. In addition, sparse data for some outcomes resulted in perfect separation (notably tobacco use by religious affiliation), leading to unstable odds ratios and wide confidence intervals and constraining inferential interpretation. These same data limitations also precluded more extensive stratified or interaction analyses, including by sex, despite the predominantly female sample, as such analyses would have produced unstable or unreliable estimates. As a result, full adjustment for all potential confounders, including age and sex, was not feasible in every multivariable model, which may have resulted in residual confounding.For some highly prevalent outcomes (e.g., physical inactivity and inadequate diet), odds ratios may overstate the magnitude of association relative to risk ratios; findings should therefore be interpreted in terms of direction and relative importance rather than precise effect size.
- Due to the cross-sectional design, temporal ordering and causal relationships between socio-demographic factors and NCD risk behaviors cannot be established.

## Conclusion

This study demonstrates that adult refugees and asylum seekers in Kalobeyei face a high and overlapping burden of modifiable and environmental risk factors for NCDs. Nearly all participants reported multiple exposures, most commonly inadequate fruit and vegetable intake, physical inactivity, high salt consumption, substance use, and dependence on polluting fuels. These risks are unevenly distributed, shaped by intersecting factors such as religious affiliation, educational level, and age. The clustering of exposures reflects broader syndemic dynamics, driven by structural vulnerability, poverty, and limited access to essential services in humanitarian settings. There is an urgent need to integrate NCD risk prevention into routine refugee health and protection programming, with a focus on structural determinants rather than individual behavior alone. Effective interventions should combine culturally sensitive health promotion, community-based education on diet and physical activity, harm reduction for substance use, and expanded access to clean energy solutions. Faith-informed, gender-responsive, and livelihood-linked approaches can offer additional, context-relevant entry points.

Ultimately, reducing NCD risk in protracted refugee settings will require coordinated, multi-sectoral action that aligns health, nutrition, energy, and protection systems, centering prevention, promoting equity, and advancing sustainable health outcomes for displaced populations.

## Data Availability

The datasets generated and/or analyzed during the current study are available from the corresponding author on reasonable request. Due to protection concerns for refugee participants, the dataset is not publicly available but may be shared upon reasonable request and subject to UNHCR/partner data-sharing approval.

## Footnotes

1 Chang’aa, a traditional brew, is a potent, locally distilled spirit widely produced in Kenya.

2 Busaa, a traditional brew, is a weaker, fermented maize or cereal beer commonly consumed in community settings in Kenya.
